# Prevalence and Species Identification of Lungworms in Sheep and Cattle: A Postmortem Study in North Shewa, Central Highlands of Ethiopia

**DOI:** 10.1155/vmi/4001491

**Published:** 2025-11-04

**Authors:** Aweke Engdawork, Bersissa Kumsa

**Affiliations:** ^1^Ethiopian Biodiversity Institute (EBI), P.O. Box 30726, Addis Ababa, Ethiopia; ^2^College of Veterinary Medicine and Agriculture, Addis Ababa University, Bishoftu, Ethiopia

**Keywords:** cattle, lungworm, North Shewa, postmortem, prevalence, sheep

## Abstract

Lungworms are among the major nematode parasites causing significant impacts on livestock production, mortality and morbidity in young animals, and poor productivity in adult animals. Despite the widespread distribution of lungworms, there is little information in North Shewa in the central highlands of Ethiopia. Thus, a cross-sectional study was conducted to determine the prevalence of lungworms, identify the major respiratory helminths, and assess putative risk factors in sheep and cattle. A postmortem examination was conducted on 400 randomly selected animals. The present study revealed an overall 20.75% (95% CI: 16.88–25.06) prevalence of lungworm infection. The prevalence of respiratory helminths was 40.69% (95% CI: 33.88–47.77) in sheep and 0% in cattle. The logistic regression analysis indicated that age and season were significant risk factors, whereas body condition was a significant predictor of lungworm infections. Young sheep were 2.3 (95% CI: 1.26–4.23; *p*=0.007) times more at risk of lungworm infection than adults. The prevalence of the disease was 4 (95% CI: 1.79–8.72; *p*=0.001) times higher in sheep with poor body conditions. The prevalence of lungworm was more than 3 (95% CI: 1.37–6.62; *p*=0.006) times higher in autumn than in spring. The most prevalent species of respiratory helminths were *Dictyocaulus filaria* (29.9%; 95% CI: 23.71–36.69), *Muellerius capillaris* (4.41%; 95% CI: 2.04–8.21), mixed infections (3.92%; 95% CI: 1.71–7.58), and *Protostrongylus rufescens* (2.45%; 95% CI: 0.80–5.63). The present study determined a higher prevalence of ovine lungworms and no evidence of the disease in cattle. The study identified age, body condition, and season as the most important risk factors for lungworm infection. Thus, regular deworming of sheep and awareness creation for the farmers on rotational and strategic grazing are forwarded to control lungworms in sheep. Moreover, further studies are required to confirm the conditions of lungworms in cattle in North Shewa.

**Summary**

• Respiratory helminths prevalence is high (41%) in sheep and absent in cattle in North Shewa in the central highlands of Ethiopia.
• Ovine lungworm prevalence correlates with the age and body condition of the animal and season of the year, indicating that lungworm prevalence is higher in young animals,animals with poor body conditions, and during wet seasons.
• *Dictyocaulus filaria* is by far the most prevalent lungworm species (29.9%), followed by *Muellerius capillaris* (4.41%), mixed infections (3.92%), and *Protostrongylus rufescens* (2.45%).

## 1. Introduction

Lungworms are a globally distributed and one of the most common parasitic diseases affecting livestock, particularly in ruminants. The disease is widely distributed in the highlands of tropical and subtropical countries and is common in Ethiopia [1]. Lungworms are nematodes that belong to the order Strongylida, infecting the respiratory system, especially the lungs of animals. The most common lungworms belong to one of two groups: the superfamily *Trichostrongyloidea* or the superfamily *Metastrongyloidea* [2]. The lungworms in the superfamily *Trichostrongyloidea* include several species of the genus *Dictyocaulus,* which infect cattle (*D. viviparus*), small ruminants (*D. filaria*), and equines (*D. arnfieldi*). *Dictyocaulus* lungworms have a direct life cycle [3, 4]. The lungworms from the superfamily of *Metastrongyloidea* include *Protostrongylidae* (*P. rufescens* and *M. capillaries*), which infect sheep and goats. *Protostrongylidae* has an indirect life cycle that involves the intermediate host (IH) of either snail or slug [2].

The epidemiological distribution of lungworms depends on several environmental and intrinsic factors. Pasture contamination by carrier animals plays a significant role in the epidemiology of lungworms. Pasture infectivity is related to rainfall, which stimulates the activity of the larvae and the mollusks [5]. The prevalence of lungworms in ruminants depends on several factors, such as climate, season, availability of IH, and favorable ecological conditions. High stocking densities increase the concentration of parasites and also force animals to graze closer to the ground [6]. Lungworms in the genus *Dictyocaulus* possess a direct life cycle. In the bronchi, adult female worms lay the larvated eggs. The eggs are coughed up and swallowed with mucus, and the first-stage larvae (L1) hatch out during their passage through the gastrointestinal tract (GIT), and L1 is excreted in feces. On pasture, the larvae molt into the second stage (L2) and develop into the infective L3 [7]. *Protostrongylus* and *Mulleries* have an indirect life cycle involving the IH of several snail and slug species [8]. Adult worms lay eggs, which are then coughed up with sputum toward the bronchi and trachea. The eggs hatched to the first larval stage in the trachea or during their passage in the GIT, and L1 are passed in the feces. Once in the environment, larvae penetrate the snails and develop into the infective L3 larval stage [9].

The pathogenesis of lungworm infection is determined by several host and parasitic factors, including age, nutritional and immunity status, degree of infestation, and predilection site in the respiratory tracts. The signs of lungworm infection range from slightly increased respiratory rates with moderate coughing to severe respiratory problems and persistent coughing [10]. Diagnosis of lungworm infection can be based on the clinical signs and grazing history of the animal. The confirmation of lungworm is by detecting the L1 larval stage in fecal samples using the Baermann technique [11]. However, the gold standard for the diagnosis of lungworm infection is postmortem examination [12]. Lungworms can be treated through broad-spectrum anthelmintic treatment such as albendazole, levamisole, and ivermectin [13, 14]. The control and prevention of lungworm can be achieved by deworming all animals at the end of the rainy season to avoid heavy parasitic burden during grazing and by deworming all animals at the end of the dry season before the rain starts to prevent or reduce pasture contamination [3]. Lungworms can also be prevented through the vaccination of larvae of *Dictyocaulus* [15].

Ethiopia has the largest livestock population in Africa, with livestock ownership currently supporting and sustaining the livelihoods of approximately 80% of the rural population. However, livestock production is characterized by lower productivity due to morbidity and mortality caused by different parasitic diseases [16]. The prevalence of lungworms in Ethiopia ranges from 13.4% to 72.4% in sheep and 1.5%–3.1% in cattle [6, 15, 17–20]. However, most lungworm studies in Ethiopia, particularly in North Shewa, were mainly based on the coprological procedure, a less sensitive technique than the gold standard postmortem technique. Moreover, there was no information available on the epidemiology of bovine lungworms in North Shewa, and only limited information was available on bovine lungworms in the country. Ethiopia is known for the presence of diverse agro-ecologies suitable for the development of lungworms and IH. Therefore, it is crucial to update the current status of lungworms to design practically appropriate control and prevention strategies. Thus, this study was designed to determine the postmortem prevalence of lungworms, identify major respiratory helminths, and assess the possible risk factors in sheep and cattle in North Shewa, in the central highlands of Ethiopia.

## 2. Materials and Methods

### 2.1. Description of the Study Area

**Figure 1:**
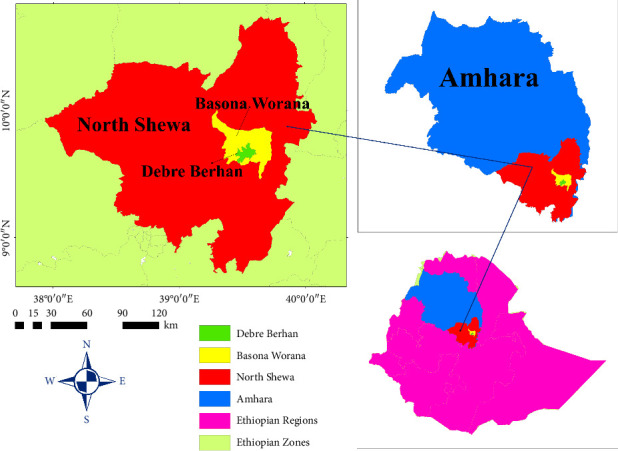
Map of the study area in the North Shewa zone of Amhara Regional State, central Ethiopia.

The study was conducted in Debre Berhan municipal abattoirs and private hotels in North Shewa in the central highlands of Ethiopia. Cattle and sheep were brought for slaughter from various areas of the North Shewa. In the study area, indigenous and crossbred cattle and sheep are the major livestock populations within a traditional mixed farming system. The North Shewa zone of the Amhara Region is one of the highest livestock-producing areas, with 1,704,407 heads of cattle and 1,941,024 sheep [21]. The area is characterized by plain grazing lands, mountain patches, and riverlines. The capital city of the North Shewa zone is Debre Berhan, a metropolitan city 130 km northeast of Addis Ababa (Figure 1). Debre Berhan is geographically located at the latitude of 9°41′N and longitude of 39°32′E with an altitude of 2840 m above sea level [22].

In North Shewa, in the central highlands of Ethiopia, the climatic condition is characterized by a long and biannually rainy season and a relatively cooler dry season. The rainy season extends from June to September, with a variable amount of rainfall in the spring. The mean annual temperature in Debre Berhan is 15.84°C, where the minimum and maximum temperatures are 6.7°C and 19.9°C, respectively. The mean annual rainfall is 1026 mm with a relative humidity of 62.3% [23]. Climatic conditions with a minimum temperature of about 10°C and a maximum temperature below 30°C are favorable to parasitic survival, development, and egg hatching [24].

### 2.2. Study Animal Populations

In this study, the cattle and sheep population located in North Shewa, particularly those brought for slaughter in Debre Berhan municipal abattoir and private hotels, were used as study animals. The study animal populations included cattle and sheep of both sexes with different ages and body condition categories. The sex, age, and body condition of all animals included in the study were recorded during antemortem inspections. The body condition scoring was classified into five scores (score 1: *thin muscle*, score 2: *moderate muscle*, score 3: *full muscle and moderate fat*, score 4: *full muscle and thick fat*, and score 5: *very full muscle and thick fat*). However, for ease of understanding, analysis, and discussion, body condition was classified into three categories: *poor* (score 2), *moderate* (score 3), and *good* (score 4 and score 5) [25]. Age estimation was based on dentition, and age was classified into young (sheep less than 1 year) and adult (sheep older than a year) [17, 19]. However, as most cattle being slaughtered reached maturity, there was no age classification [26]. The standard postmortem examinations were conducted on selected cattle and sheep slaughtered at Debre Berhan municipal abattoir and private hotels [27].

### 2.3. Sampling Methodology and Sample Size Determination

The present study employed a stratified random sampling technique to select the study animals and collect data relevant to the abattoir survey. The variable species was used for the stratification of the study animal populations. The study animals were stratified as cattle and sheep, and a simple random sampling technique was used to select individual animals in each stratum. The study gives special emphasis on the investigation of animals brought from grazing. The sample size for the study was determined using the standard formula described by Thrusfield [28]. The desired sample size was calculated based on a 95% confidence interval (CI) and 5% desired absolute precision. Herewith, there were no previous studies that established the prevalence and putative risk factors of lungworm infections in cattle in North Shewa in the central highlands of Ethiopia. Accordingly, there were no previous studies based on the postmortem examination of ovine lungworm infection. Therefore, the sample size was determined using an expected prevalence of 50% lungworm infection [29]. Accordingly, the minimum sample required for this study was 384 animals. However, in this study, 400 animals (196 cattle and 204 sheep) were sampled to increase the precision of the study.

### 2.4. Study Design and Sample Collection

The present study employed a cross-sectional investigation on the prevalence of lungworm infections in cattle and sheep. The proper antemortem and postmortem examinations were conducted in the study for the ruminant species. Antemortem procedures include the stratification of animal populations; random selection of study animals; detailed recording of animal species, sex, age, and body condition scores of the animal; and season of the year. Postmortem examinations were conducted with standard procedures to determine the status of lungworm infection, the degree of parasitic infestation (worm load), and the species of respiratory helminths. During antemortem inspections, each of the study animals was given an identification number by a paint mark on their body, and information was collected on the putative risk factors.

### 2.5. Postmortem Examinations

Standard postmortem examinations were conducted for each sampled animal by taking the lungs immediately following slaughter. The lungs were examined first by visual inspection and palpation. Then, finally, systemic incisions of the lungs were conducted to appreciate the presence of lungworms, the degree of parasitic infestation, and the species of lungworms [30]. Each lung from the study animals was inspected by incising it starting from the trachea down to the bronchi and bronchioles and then making multiple deep incisions of the lobes with many small subcuts. The recovered worms were kept in 70% alcohol and transferred to the laboratory for examination. Adult parasites were examined under the stereomicroscope to determine the degree of infestation or worm burden and the identification (classification) of adult lungworm parasites at the species level [15, 31].

### 2.6. Data Management and Statistical Analysis

The data generated from antemortem and postmortem examinations and laboratory findings were entered into a Microsoft Excel spreadsheet and summarized using descriptive statistics. The many attribute data imported to the database system include host risk factors, such as species, sex, age, and body condition of the sampled animals; the season of the year; and laboratory results of the degree of parasitic infestation and lungworm species. This study employed STATA Statistical software, Version 14.0, for the statistical analysis of risk factors. The prevalence of lungworms was determined using descriptive statistics. The associations of lungworm infections with putative risk factors and predictor variables were determined using logistic regression analysis. The correlation of the degree of parasitic burden with body condition categories was indicated by a distributional graphics plot. The hypothesized risk factors were considered statistically significant factors of the diseases when the *p* value is less than 0.05 at a 95% CI.

## 3. Results

### 3.1. Prevalence of Lungworm Infections

In the present study, among 400 animals examined for the presence of lungworms, 83 animals were found positive for lungworm infection. Therefore, the overall prevalence of lungworms in North Shewa in the central highlands of Ethiopia was 20.75% (95% CI: 16.88–25.06) based on postmortem examinations. Among 204 sheep examined for lungworms, 83 animals (40.69%; 95% CI: 33.88–47.77) were found positive. Out of 196 cattle sampled for postmortem lungworm examinations, no animal was found positive for lungworm infection. The current results indicated that there was a significant difference (OR = 270.09; 95% CI: 16.60–4394.35; *p*=0.001) in the prevalence of lungworm infections between cattle and sheep (Table 1).

### 3.2. Prevalence of Ovine Lungworms and Putative Risk Factors

The present study revealed that the prevalence of lungworms was 41.23% in male animals and 40% in females. However, there was no significant difference in the prevalence of the disease with the sex of the animal (*p* > 0.05). The univariable logistic regression analysis indicated that the age factor was significantly associated with lungworm infections. Young animals were 2.3 (95% CI: 1.26–4.23; *p*=0.007)times more at risk of lungworms than adult sheep. The prevalence of lungworms was 58.06%, 37.5%, and 25.93% in sheep with poor, moderate, and good body conditions, respectively. The results stated that sheep with poor body conditions were nearly 4 (OR = 3.96; 95% CI: 1.79–8.72; *p*=0.001) times more infected with lungworms than sheep with good body conditions. The present study indicated that the season of the year was a significant factor in lungworm infections. The prevalence of the disease was 51.43% in autumn, 40.48% in winter, and 26% in spring. The prevalence of lungworms was 3 (OR = 3.01; 95% CI: 1.37–6.62; *p*=0.006) times higher in autumn than in the spring season (Table 2).

### 3.3. Degree of Parasitic Infestation

**Figure 2:**
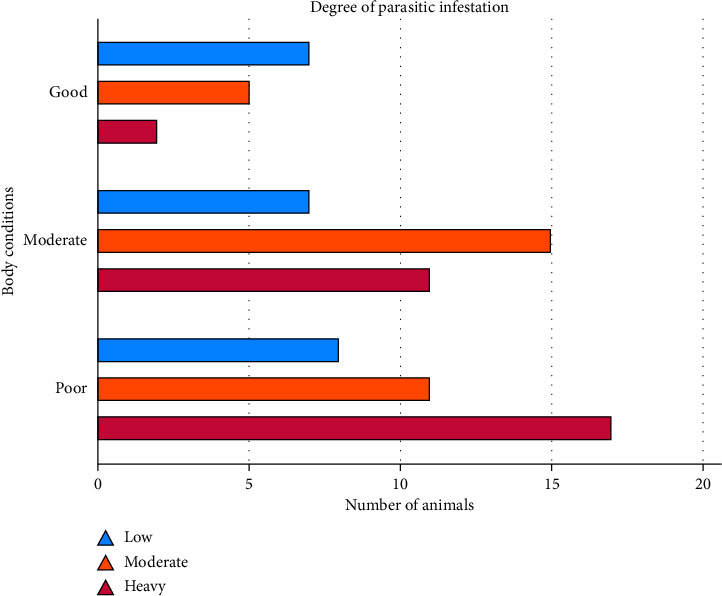
Graphical presentation of the association of the degree of parasitic infestation with body condition of the animal.

Besides determining the prevalence of lungworm infections and their association with putative risk factors, the present study also indicated the degree of parasitic infestation (parasitic burden). The current results revealed that most lungworm-infected sheep had a higher parasitic burden. Accordingly, 36.14% of infected sheep had a heavy parasitic infestation, 37.35% had a moderate parasitic burden, and only 26.51% of infected sheep had a low degree of parasitic infestation. The present study showed significant differences in the degree of parasitic infestation with the body conditions of the animals. The degree of parasitic infestation (lungworm burden) increased as the body condition of the animal getting worse (Figure 2). The results indicated that most lungworm-infected sheep with a heavy degree of parasitic infestation (47.22%) possessed poor body conditions. The present findings also revealed that most sheep with good body conditions (50%) were infected with a low degree of parasitic burden (Table 3).

### 3.4. Species of Ovine Respiratory Helminths

The present study indicated the presence of various lungworm species and mixed infections in sheep. The most prevalent ovine respiratory helminths were *D. filaria* (29.9%; 95% CI: 23.71–36.69), *M. capillaris* (4.41%; 95% CI: 2.04–8.21), *P. rufescens* (2.45%; 95% CI: 0.80–5.63), and mixed infections (3.92%; 95% CI: 1.71–7.58). *D. filaria* was by far the most prevalent lungworm species of sheep in North Shewa in the central highlands of Ethiopia. The results revealed that the prevalence of *D. filaria* was nearly 17 (OR = 16.98; 95% CI: 6.65–43.32; *p* = 0.001) times higher than that of *P. rufescens*. Mixed infections with dual or triple lungworm species where coinfections of* D. filaria* with* P. rufescens *and/or* M. capillaris* and coinfections of *P. rufescens* with *M. capillaris* were observed (Table 4).

## 4. Discussion

The present study revealed a higher prevalence of lungworm infections in sheep in North Shewa in the central highlands of Ethiopia. The prevalence of the disease was 40.69% (95% CI: 33.88–47.77) based on postmortem examinations. The prevalence of ovine lungworms in the present study was in close agreement with the previous studies that reported a lungworm prevalence of 43.3% in Dessie Zuria [32], 42.0% in North Gondar Zone [33], and 44.02% in Durame district, southern Ethiopia [15]. However, the finding of the present study was higher than that reported a lungworm prevalence of 18.2% in and around Bahir Dar [34], 8.6% in Mekedella district [35], and 20.2% in Bahir Dar district [36]. The prevalence of ovine lungworms in the current study was lower than the studies that reported the prevalence of 57.1% in the Tiyo district [37], 56.3% in North Shewa [29], and 66.3% in northeastern Ethiopia [38]. The variations in the prevalence of lungworms in sheep could be attributed to the differences in the sample sizes, study areas, seasons of the year, and methods of examinations employed in the studies. The present study reported the results of the postmortem examination of the lungs, the most sensitive and specific, and the gold standard method of diagnosis for lungworms.

In the present study area, there was no evidence of lungworm infection in cattle. Similar to the present finding, the absence of lungworms was reported in cattle in the Kirikkale province of Turkey [39], whereas a lower prevalence of 0.5% was reported in the Addis Ababa abattoir [26]. However, other findings indicated relatively higher prevalence of 1.5% in Addis Ababa abattoir [19], 3.1% in Gondar [20], and 3.98% in southern Ethiopia [15]. The absence of lungworms in cattle in the present finding could suggest the use of broad-spectrum anthelmintics in fattening cattle to get the possible maximum weight gain that significantly reduces the chance of lungworms [40, 41]. Moreover, cattle slaughtered at Debre Berhan municipal abattoir were mostly brought from feedlots, which had a lower exposure to the disease.

The univariable logistic regression analysis indicated a significant difference in the prevalence of lungworms between cattle and sheep (OR = 270.09; 95% CI: 16.60–4394.35; *p* = 0.001). In line with the present finding, a significant difference was also reported in the Durame district [15]. The use of broad-spectrum anthelmintics to get the maximum possible weight gain in feedlot cattle is cited as the major justification behind the finding. There was no significant difference in the prevalence of lungworms between male and female sheep. Accordingly, insignificant associations were reported in North Shewa [29] and Wolaita Sodo [1]. In contrast to the present finding, studies conducted around Bahir Dar city [36] and in Legambo district [38] reported a significant association of lungworm infection with sex groups. The differences in the findings are majorly attributed to the variations in sampling and sample sizes employed in the studies.

The finding of a significant association (*p* < 0.05) between lungworm infection and age groups in this study was in line with several findings across Ethiopia [19, 38, 42]. In contrast, some studies [43, 44] reported insignificant differences between age groups. The difference in the findings might be due to the variations regarding the cut point for age classifications of sheep or other unforeseen factors. Young animals are generally susceptible to parasitic infection due to an immature immune system. The significant difference in the prevalence of lungworms among the seasons of the year in this study was similar to the findings of several studies [5, 17, 45]. The highest prevalence of lungworm infection was observed in wet seasons (autumn). The findings suggest that a damp and cool environment is very suitable for the development of lungworms and infectivity of the third-stage larva (L3) in sheep.

The present study revealed a higher prevalence of lungworm in sheep with poor body conditions. Similarly, several findings [42, 46–48] indicated a significant variation in the prevalence of lungworm with body conditions. This might be attributed to the nutritional status of the animals and concurrent infections. The parasitic resistance and resilience abilities of the animal are significantly influenced by nutrition, and poor nutrition lowers immunity and tolerance to clinical signs. This enhances the establishment of worms and increases the prevalence in sheep with poor body conditions [49, 50]. Accordingly, the heaviest degree of parasitic infestation (worm load) in animals with poor body conditions in this study was in line with several findings [15, 16]. The possible explanation for the largest worm burden in sheep with poor body conditions could be an increase in the degree of pasture contamination in the extensive system of production and less completeness of poorly nourished animals in getting rid of lungworms. Well-fed animals usually succumb to the disease when there are the right environmental conditions.

*D. filaria* was the dominant species of respiratory helminths in the present study areas. Many other previous studies [1, 6, 18, 51, 52] also reported *D. filaria* as the predominant species circulating in Ethiopian sheep and goats managed under traditional husbandry systems. In contrast, other studies [17, 38] reported the preponderance of small lungworms over *D. filaria* infection. The lower prevalence of small lungworms in the present study area could be associated with their complex life cycle. Unlike *D. filaria*, which has a direct life cycle, *P. rufescens* and *M. capillaris* have an indirect life cycle requiring a molluscan IH to complete their development [13]. The climatic conditions in the area, as well as the seasons of the study, might not be conducive for the survival and breeding of the IHs that lowered the prevalence of small lungworm species [53].

## 5. Conclusions

The present study revealed a higher prevalence of lungworm infection in sheep and no evidence of the disease in cattle. The study identified that age groups, body conditions, and seasons of the year were significantly associated with ovine lungworm infection. The prevalence of lungworm infection was higher in young sheep, in sheep with poor body condition, and during the autumn season. Accordingly, the degree of parasitic infestation worsens in animals with poor body condition. The study revealed that *D. filaria* was the dominant species of respiratory helminths in the study area. The findings of this study indicated that lungworm is one of the common health problems and productivity constraints of sheep in North Shewa, the central highlands of Ethiopia. Thus, this study recommends regular sheep deworming and awareness creation for the farmers on rotational and strategic grazing to control ovine lungworm infection, thereby improving sheep production and productivity. Moreover, further investigations are suggested to confirm the actual situation of lungworms in cattle and other domestic animals in Ethiopia.

## Figures and Tables

**Table 1:** The prevalence of lungworm infections in sheep and cattle in North Shewa in the central highlands of Ethiopia.

Species	Total examined	No. of positives	Prevalence (95% CI)	OR (95% CI)	*p* value
Sheep	204	83	40.69 (33.88–47.77)	270.09 (16.60–4394.35)	0.001
Cattle	196	0	0.00 (0.00–1.86)	1	
**Total**	**400**	**83**	**20.75 (16.88–25.06)**		

*Note:* 1, reference. The bold values indicate the total number of positive animals and the overall prevalence of lungworm infections.

Abbreviations: CI, confidence interval; OR, odds ratio.

**Table 2:** Univariable logistic regression analysis of ovine lungworm infection and associated risk factors.

Predictive variables	No. of animals examined	No. of positives	Prevalence	OR (95% CI)	*p* value
*Sex*					
Male	114	47	41.23	1.05 (0.60–1.85)	0.859
Female	90	36	40.00	1	

*Age*					
Adult	142	49	34.51	1	
Young	62	34	54.84	2.30 (1.26–4.23)	0.007

*Body condition*					
Good	54	14	25.93	1	
Moderate	88	33	37.50	1.71 (0.81–3.62)	0.157
Poor	62	36	58.06	3.96 (1.79–8.72)	0.001

*Season of the year*					
Autumn	70	36	51.43	3.01 (1.37–6.62)	0.006
Winter	84	34	40.48	1.94 (0.90–4.17)	0.092
Spring	50	13	26.00	1	

*Note:* 1, reference.

Abbreviations: CI, confidence interval; OR, odds ratio.

**Table 3:** Degree of ovine lungworm infestation and its association with body condition of the animal.

Degree of lungworm infestation	No. of animals	Proportion	Body condition classification		
			Poor (%)	Moderate (%)	Good (%)
Low	22	26.51	8 (22.22)	7 (21.21)	7 (50.00)
Moderate	31	37.35	11 (30.56)	15 (45.46)	5 (35.71)
Heavy	30	36.14	17 (47.22)	11 (33.33)	2 (14.29)
**Total**	**83**	**100**	**36 (100)**	**33 (100)**	**14 (100)**

*Note:* The bold values indicate the total number of positive sheep.

**Table 4:** The species of major ovine respiratory helminths and their prevalence in the study areas.

Lungworm species	No. of positives	Prevalence (95% CI)	OR (95% CI)	*p* value
*Dictyocaulus filaria*	61	29.90 (23.71–36.69)	16.98 (6.65–43.32)	0.001
*Muellerius capillaris*	9	4.41 (2.04–8.21)	1.84 (0.60–5.58)	0.283
*Protostrongylus rufescens*	5	2.45 (0.80–5.63)	1	
Mixed infection	8	3.92 (1.71–7.58)	1.62 (0.52–5.05)	0.402
**Total**	**83**	**40.69 (33.88–47.77)**		

*Note:* 1, reference. The bold values indicate the total prevalence of respiratory helminths in sheep.

Abbreviations: CI, confidence interval; OR, odds ratio.

## Data Availability

The data that support the findings of this study are available from the corresponding author upon reasonable request.

## References

[1] Abebe R., Melesse M., Mekuria S. (2016). Lungworm Infection in Small Ruminants in and Around Wolaita Soddo Town, Southern Ethiopia. *Journal of Veterinary Science & Technology*.

[2] Mandal S. (2006). *Veterinary Parasitology at a Glance*.

[3] Tewodros A. (2015). A Review On: Lungworm Infection in Small Ruminants. *World Journal of Pharmaceutical and Life Sciences*.

[4] Jemal A. (2016). Lungworm Infection of Small Ruminant in Ethiopia: A Review. *World Journal of Pharmaceutical and Life Sciences*.

[5] Borji H., Azizzadeh M., Ebrahimi M., Asadpour M. (2012). Study on Small Ruminant Lungworms and Associated Risk Factors in Northeastern Iran. *Asian Pacific Journal of Tropical Medicine*.

[6] Kebede S., Menkir S., Desta M. (2014). On Farm and Abattoir Study of Lungworm Infection of Small Ruminants in Selected Areas of Dale District, Southern Ethiopia. *International Journal of Current Microbiology and Applied Sciences*.

[7] Shite A., Admassu B., Yenew A. (2015). Bovine Dictyocaulosis: A Review. *European Journal of Biological Sciences*.

[8] Kahn M. (2005). *The Merck Veterinary Manual*.

[9] Junquera P. (2015). Protostrongylus Rufescens, Parasitic Lungworms of Sheep and Goats. *Biology, Prevention and Control. Protostrongylosis*.

[10] Elsheikh H., Khan N. (2011). *Essentials of Veterinary Parasitology*.

[11] Hasen A., Takele S., Simenew K. (2013). Ovine Lungworm Infestation Rate on Fecal Larvae Recovery Basis. *Acta Parasitologica Globalis*.

[12] Fentahun S., Abebe R., Melkamu S., Asrat M. (2016). Study on Lungworm Infection in Small Ruminants: Prevalence and Risk Factors in and Around Gondar Town, Northwest, Ethiopia. *International Journal of Veterinary Sciences and Animal Husbandry*.

[13] Taylor M., Coop R., Wall R. (2007). *Veterinary Parasitology*.

[14] Lefevre P., Jean B., Ren C., Gerrit U. (2010). Infectious and parasitic diseases of livestock.

[15] Fesseha H., Mathewos M. (2021). Prevalence and Risk Factors of Bovine and Ovine Lungworm Infection at Durame District, Southern Ethiopia. *Journal of Parasitology Research*.

[16] Asaye M., Alemneh T. (2015). Prevalence of Lungworm Infection of Small Ruminants in and Around Bahir Dar City, Amhara Regional State, Ethiopia. *Journal of Veterinary Science & Technology*.

[17] Regassa A., Toyeb M., Abebe R. (2010). Lungworm Infection in Small Ruminants: Prevalence and Associated Risk Factors in Dessie and Kombolcha Districts, Northeastern Ethiopia. *Veterinary Parasitology*.

[18] Fentahun T., Seifu Y., Chanie M., Moges N. (2012). Prevalence of Lungworm Infection in Small Ruminants in and Around Jimma Town, Southwest Ethiopia. *Global Veterinaria*.

[19] Zarihun W., Tesfaye M. (2016). Study on Comparative Prevalence of Lungworms of Sheep and Cattle Slaughtered at Addis Ababa Abattoir, Ethiopia. *International Journal of Research Studies in Biosciences (IJRSB)*.

[20] Awake M., Debeb D. (2017). Study on the Prevalence of Bovine Lungworm in Gondar Town, North Ethiopia. *International Journal of Advanced Research*.

[21] CSA (2021). Federal Democratic Republic of Ethiopia Central Statistical Agency Agricultural Sample Survey 2021 Volume II Report on Livestock and Livestock Characteristics. *Central Statistical Agency Statistical Bulletin 589. Addis Ababa, Ethiopia*.

[22] Fikire A. H. (2021). Determinants of Urban Housing Choice in Debreberhan Town, North Shewa Zone, Amhara Region, Ethiopia. *Cogent Economics & Finance*.

[23] Lakew A., Goshu G., Mamo G., Mengistuand A., Demissie T. (2022). Sero-Prevalence of Bovine Brucellosis in Selected Dairy Farms of Debreberhan Milkshed, Central Highlands of Ethiopia. *Global Veterinaria*.

[24] Deressa T., Birhan W., Enawgaw B. (2018). Proportion and Predictors of Transfusion-Transmissible Infections Among Blood Donors in North Shewa Zone, Central North Ethiopia. *PLoS*.

[25] Kenyon P. R., Maloney S. K., Blache D. (2014). Review of Sheep Body Condition Score in Relation to Production Characteristics. *New Zealand Journal of Agricultural Research*.

[26] Fekadu S. (2008). *A Study on Common Gross Lungs of Cattle Slaughtered at Addis Ababa*.

[27] Gebru L. (2008). Epidemiology and Economic Impacts of Fasciolosis of Domestic Ruminants in Selected Sites of Tigray Regional State, Northern Ethiopia. *CVMA, AAU, Debre Zeit*.

[28] Thrusfield M. (2018). *Veterinary Epidemiology and Surveys (Modern Fou)*.

[29] Tefera Y., Mekuria S. (2016). Lungworm in Ovine: Prevalence and Associated Risk Factors in Debre Berhan Town, Ethiopia. *Journal of Veterinary Science & Technology*.

[30] Pereckienė A., Kaziūnaitė V., Vyšniauskas A. (2007). A Comparison of Modifications of the McMaster Method for the Enumeration of Ascarissuum Eggs in Pig Faecal Samples. *Veterinary Parasitology*.

[31] Umur S., Koroglu E., Guclu F., Tinar R. (2006). Nematode in Helminthology.

[32] Basaznew B., Ayalew E., Achenef M. (2012). Ovine Lungworm Infection: Prevalence, Species Composition and Associated Risk Factors in Dessie Zuria District, Northeastern Ethiopia. *African Journal of basic Applied Science*.

[33] Yitagel T., Tafess K., Fekadie G., Kebede N. (2013). Prevalence of Lungworm Infection in Small Ruminants in North Gondar Zone, Amhara National Regional State, Ethiopia. *Journal of Parasitology and Vector Biology*.

[34] Muluken Y. (2009). *Prevalence of Ovine Lungworms in and Around Bahirdar. DVM Thesis, College of Agriculture and Veterinary Medicine, School of Veterinary Medicine*.

[35] Gebreyohannes M., Alemu T., Kebede E. (2013). Prevalence of Ovine Lungworms in Mekedella Woreda, Ethiopia. *Journal of Animal Production Advances*.

[36] Kassa T., Abidu M. (2013). Prevalence of Ovine Lung Worms-Around Bahir Dar, East Africa, Ethiopia. *Acta Parasitol Global*.

[37] Bekele M., Abu A. (2011). Ovine Lungworms in Tiyo District, South-East Ethiopia: Prevalence, Effect of Altitude and Major Host Related Risk Factors. *Global Veterinaria*.

[38] Alemu S., Leykun E., Ayelet G., Zeleke A. (2006). Study on Small Ruminant Lungworms in Northeastern Ethiopia. *Veterinary Parasitology*.

[39] Kader Y. (2006). Prevalence of Lungworm Infection in Sheep and Cattle in the Kirikkale Province, Turkey. *Eparazitoloji Dergisi*.

[40] Lat-Lat H., Hassan L., Sani R., Sheikh-Omar A., Hishamfariz M., Ng V. (2007). First Report of Bovine Lungworm Disease in South-East Asia. *Tropical Biomedicine*.

[41] Nishimiyimana J., Shyaka A., Mutandwa E. (2010). Effect of Altitude and Animal Age on the Prevalence of Dictyocaulosis in Cattle in the Northen Province of Rwanda. *Journal of Agricultural Extension and Rural Development*.

[42] Teshome Z., Woldehana T. (2020). Prevalence of Ovine Lung Worm in and Around Assela. *International Journal of Advanced Research in Biological Sciences*.

[43] Mesfin N. (2008). Study on Prevalence of Small Ruminant Lungworm in and Around Kombolcha, South Wollo, Ethiopia. *DVM Thesis*.

[44] Hassan A., Mezgebu E. (2023). Prevalence of Lungworm Infection of Small Ruminants in and Around Sebeta Town, Central Ethiopia. *Austin Journal of Veterinary Science & Animal Husbandry*.

[45] Mulate B., Mamo M. (2016). Prevalence and Financial Losses of Lungworm Infection in Sheep in South Wollo Zone, Ethiopia. *Journal of Animal Research*.

[46] Eyob E., Matios L. (2013). The Prevalence and Risk Factors Associated With Ovine Lungworm Infestation in the Asella Province, Central Ethiopia. *Journal of Parasitology and Vector Biology*.

[47] Dessalew H., Ananiya S. (2019). Prevalence, Associated Risk Factors and Species Identification of Lung Worm Infection in Sheep in Dangla District, Western Amhara, North West Ethiopia. *International Journal of Veterinary Sciences Research*.

[48] Abdeta D., Degefa Y. (2020). Prevalence of Ovine Lung Worm and Associated Risk Factors in Honkolowabe District, East Arsi Zone, Ethiopia. *Biomedical Journal of Scientific & Technical Research*.

[49] Walkden-Brown S., Kahn L. (2002). Nutritional Modulation of Resistance and Resilience to Gastrointestinal Nematode Infection: A Review. *Asian-Australasian Journal of Animal Sciences*.

[50] Lamidi A., Aina A., Alikwe P. (2013). Digestibility and Nitrogen Balance of Sole Malted Sorghum Sprout, Maize Stover and Rice Straw in West African Dwarf Goat. *African Journal of Livestock Extension*.

[51] Moges N., Bogale B., Chanie M. (2011). Dictyocaulus Filaria and *Muellerius capillaris* Are Important Lungworm Parasites of Sheep in Wogera District, Northern Ethiopia.

[52] Addis M., Fromsa A., Ebuy Y. (2011). Study on the Prevalence of Lungworm Infection in Small Ruminants in Gondar Town, Ethiopia. *Journal of Veterinary Research*.

[53] Dessalew H., Ananiya S. (2019). Prevalence, Associated Risk Factors and Species Identification of Lung Worm Infection in Sheep in Dangla District, Western Amhara, North West Ethiopia. *International Journal of Veterinary Sciences Research*.

